# Safety and Influenza Infections in Children Aged 6–35 Months Receiving Cell Culture-Derived Inactivated Quadrivalent Influenza Vaccine During the 2023–2024 Influenza Season in South Korea

**DOI:** 10.3390/vaccines13050501

**Published:** 2025-05-08

**Authors:** Hye Eun Lee, Seong-Beom Park, Hye-Young Kim, Sun Heom Baik, Kyungyeon Jung, Juhwan Kim, Ji Young Park

**Affiliations:** 1 Medical Affairs, SK bioscience Co., Ltd., Seongnam 13494, Republic of Korea; skydms29@sk.com (H.E.L.); hykim620@sk.com (H.-Y.K.);; 2 Department of Biohealth Regulatory Science, Sungkyunkwan University, Suwon 16419, Republic of Korea; 3 School of Pharmacy, Sungkyunkwan University, Suwon 16419, Republic of Korea; 4 Department of Pediatrics, Korea University Ansan Hospital, Ansan 15355, Republic of Korea

**Keywords:** influenza infections, child, influenza, human, influenza vaccines, safety

## Abstract

Background/Objectives: Influenza poses a significant risk for young children, particularly those under five. Cell culture-derived influenza vaccines offer advantages in reducing adaptive changes and mitigating egg allergy concerns. SKYCellflu^®^ quadrivalent has been in use since 2015, and this study aimed to assess its safety and influenza infections in children aged 6–35 months in South Korea. Methods: A prospective cohort, non-interventional, multi-center post-marketing surveillance study was conducted from 2020 to 2024. This study presents data from the 2023–2024 influenza season on safety and influenza infections in children aged 6–35 months following SKYCellflu^®^ vaccination. Safety was assessed based on adverse events (AEs) within 28 days post-vaccination, and influenza infections were assessed via phone calls or medical record screening. Results: Among 333 safety set participants, 54.4% reported at least one AE, with most being mild to moderate. The cumulative incidence of influenza infections among 247 ad hoc subsets was 4.5%, and the incidence rate was 1.3 per 100 person-months (95% CI, 0.7–2.4) during the 2023–2024 influenza season. The two-dose regimen in vaccine-naïve infants aged 6–11 months showed a lower cumulative incidence of influenza infection rate (0.8% vs. 3.8%) and incidence rate (0.3 vs. 0.9 per 100 person-months) than the one-dose group (3.8%). No influenza-related hospitalizations occurred within the ad hoc subset. Conclusions: This study demonstrated a tolerable safety profile and the pattern of influenza infections following SKYCellflu^®^ vaccination. Additionally, the two-dose regimen was associated with a lower incidence of influenza infections, suggesting potential benefits in enhancing protection among infants aged 6–11 months.

## 1. Introduction

Seasonal influenza virus is a major pathogen that causes significant morbidity and mortality worldwide, leading to one billion cases of seasonal influenza annually, including 3–5 million cases of severe illness. Children under 5 years of age are at significant risk for seasonal influenza virus, and nearly 99% of deaths due to influenza-related lower respiratory tract infections occur in developing countries [1]. Furthermore, children younger than 5 years old, especially those younger than 2 years old, are at an increased risk of developing serious flu-related complications. Notably, about 80% of reported flu-related deaths in children occurred in children who were not fully vaccinated [2,3,4].

This significant burden of seasonal influenza in children can be explained by differences in their innate and adaptive immune responses. Specifically, newborns, being almost entirely naïve to influenza, may struggle with viral clearance due to insufficient production of IFN-γ and IL-12 by the innate immune system. Additionally, the upregulation of IL-6 can lead to excessive and harmful inflammation [5]. Thus, these clinical and immunological factors contribute to a higher risk against the influenza virus, as it is challenging for children to mount an adequate response against the virus throughout their natural life course. Furthermore, children frequently interact with individuals outside the household in close-quarter locations, such as schools and day-care centers, creating an environment where influenza can quickly propagate through a pediatric population [4]. Consequently, the implementation of effective vaccination strategies for children is critical to mitigating the spread and burden of influenza.

In light of these scientific backgrounds, South Korea implemented the national immunization program (NIP) against influenza for children aged 6 months to 13 years, pregnant women, and the elderly (65 and older). Particularly, to reduce the high burden of influenza in children, two doses of the influenza vaccine are generally recommended for children under 8 years old who are receiving the influenza vaccine for the first time, to ensure an adequate immune response [3]. During the 2023–2024 season, the NIP achieved a high level of vaccination coverage, with 69.5% for children and 82.5% for the elderly [6].

Developed in South Korea, SKYCellflu^®^—the first cell culture-derived inactivated quadrivalent influenza vaccine, was designed to address several limitations of traditional egg-based vaccines, such as lengthy production times, the risk of egg protein allergies, hemagglutinin mutations, and difficulties in mass production and was authorized by the Ministry of Food and Drug Safety (MFDS) on 24 December 2015. Since its approval, the indications have expanded, and it is now approved for use in individuals aged from 6 months to adults. The vaccine is currently authorized in 11 countries and one international organization.

As part of the regulatory requirements for the age expansion of the vaccine in South Korea, a post-marketing surveillance (PMS) study has been conducted on children aged 6–35 months since 2020. Our study aimed to assess the safety profile of SKYCellflu^®^ in children aged 6 to 35 months, and as an extension of the PMS study, evaluate the incidence of influenza infections after vaccination during the 2023–2024 influenza season.

## 2. Materials and Methods

### 2.1. Study Design and Data Source

We conducted a prospective cohort, non-interventional, single-arm, multi-center, PMS study of SKYCellflu^®^ in South Korea from 12 June 2020 to 11 June 2024 at 23 study sites, with children aged 6 to 35 months. The primary objective was to assess vaccine safety by monitoring adverse events (AEs) within 28 days post-vaccination, with data collected through phone calls, electronic medical record (EMR) screening, or site visits.

The study began during the COVID-19 pandemic, coinciding with the 2020–2021 influenza season. However, a rapidly changing clinical situation and an evolving public health response posed significant challenges to its progress. Consequently, the study was suspended during the pandemic and restarted in the 2023–2024 influenza season, with operational modifications compared to the previous study period. As this was the final season for participant recruitment, a site management organization (SMO) was deployed to support study-related activities and ensure the active collection of safety data and influenza cases. In addition to the standard 28-day safety follow-up period mandated by the original PMS study, the follow-up duration was extended to 1–5 months for an ad hoc analysis of influenza infections in the 2023–2024 influenza season, as an exploratory measure to gather additional information. These operational changes may help explain the differences observed in seasonal data, which will be detailed in subsequent sections.

This study received approval from the Institutional Review Board (IRB) of Chung-Ang University Hospital (IRB no. 2011-020-19343) and Sungkyunkwan University (IRB no. 2025-02-11).

### 2.2. Study Population

The study population consisted of children aged 6 to 35 months who were eligible for SKYCellflu^®^ manufactured by SK bioscience Co., Ltd. vaccination according to the local prescribing information [7]. Children receiving the SKYCellflu^®^ vaccine for the first time were included, while those with contraindications according to the local prescribing information or the judgment of the physician were excluded from the study. In accordance with local regulatory guidelines, a total of 616 children were enrolled in the study during the 2020–2021 and 2023–2024 influenza seasons for safety evaluation. Of these, 612 participants were eligible for safety evaluation, with 338 participants identified across 12 sites during the 2023–2024 season. Five participants who received heterologous vaccination were excluded from the analysis. Consequently, the safety set consisted of 333 participants. Parents or legal representatives of participants were required to sign an informed consent form and ensure compliance with the study protocol, including completing diary cards and attending follow-up visits. The enrolled participants received the SKYCellflu^®^ vaccine, which was a part of the NIP in South Korea. The vaccine was distributed as part of routine immunization to children aged 6 months to 13 years during the 2023–2024 influenza season.

**Figure 1:**
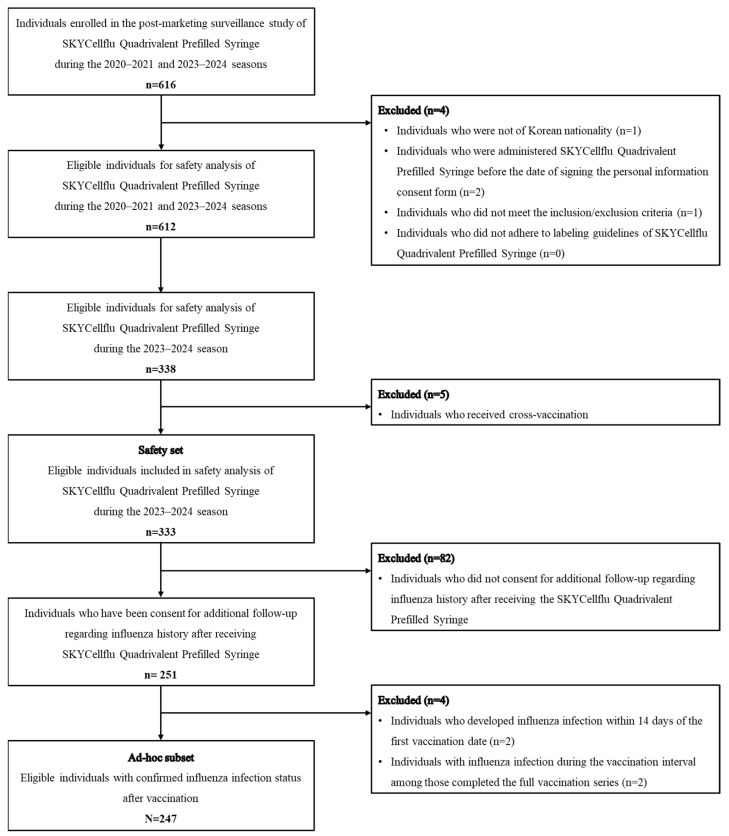
Flow chart of study population.

Of the 333 participants enrolled in the 2023–2024 influenza season, the ad hoc subset consisted of 247 children who participated in the extended follow-up to assess the incidence of influenza infections. Two participants who experienced a influenza infection within 14 days post-vaccination were excluded from the ad hoc analysis, considering that 10–14 days are required after vaccination to mount an optimal immune response and protection [8]. Among participants who completed the full vaccination series (two dose regimen), two participants who developed influenza infections before receiving the second dose were excluded from the analysis. A flow diagram of the study population is provided in Figure 1.

### 2.3. Study Outcome

The primary outcome of the study, as defined in the original PMS protocol, was vaccine safety. AEs occurring within 28 days post-vaccination were collected through phone calls, EMR screening, or site visits. For participants who received two doses, AEs were recorded within 28 days after each dose. Consequently, the total safety follow-up duration varied for each participant, depending on whether they received one or two doses. The collected AEs were evaluated by investigators in accordance with the MFDS guideline during the study and were coded using MedDRA version 27.0. The safety endpoints included the overall incidence of AEs, adverse drug reactions (ADRs), and serious adverse events (SAEs). Furthermore, the severity of AEs was classified into three categories: mild, moderate, and severe. AEs were considered mild if they were easily tolerated and did not interfere with daily activities, moderate if they caused discomfort and interfered with daily activities, and severe if they prevented normal daily activities, according to the PMS guideline from the MFDS. Furthermore, solicited AEs, defined as AEs systematically collected during the 7-day post-vaccination period, were also evaluated.

For the ad hoc analysis, the cumulative incidence and incidence rate of influenza infection was estimated based on the last vaccination. Influenza infections were defined as laboratory-confirmed cases of influenza or clinically diagnosed influenza cases, verified either through phone calls or EMR screening occurring 14 days post-vaccination.

Regarding phone calls, participants were eligible to receive up to three phone call attempts if they did not respond to the initial contact. In cases of non-response, a short text message service was utilized to encourage data collection. The response rate for the additional follow-up phone survey was 99.2%. Furthermore, the cumulative incidence of hospitalization in influenza cases among ad hoc subset was estimated to assess the burden and severity of influenza disease.

### 2.4. Statistical Analysis

Safety and influenza infection analyses were conducted separately for participants enrolled in the 2023–2024 influenza season. The safety analysis was conducted based on 333 participants who completed the 28-day follow-up period after receiving at least one dose of SKYCellflu^®^ vaccine, while the ad hoc analysis was performed on a subset of 247 participants who completed an additional 1 to 5 months of follow-up beyond the initial period. Baseline characteristics including age, sex, number of doses, and pediatric comorbidity index of the safety set and ad hoc set were summarized using descriptive statistics [9]. Continuous variables were summarized as means with standard deviations (SD), and categorical variables were described as frequencies with percentages.

The incidence of AEs, ADRs, and SAEs was calculated with 95% confidence intervals (CIs) by using the exact binomial distribution. The incidence of solicited AEs was also calculated. Subgroup analyses were performed based on the number of doses received, categorizing participants into a one-dose group and a two-dose group. The two-dose group was further analyzed according to the order of doses—Dose 1 after the first dose and Dose 2 after the second dose—applying a uniform 28-day post-vaccination period for each dose [10].

The cumulative incidence of influenza infections from September 2023 to March 2024 was calculated. For each month, the cumulative incidence was calculated by dividing the number of new influenza infection cases by the number of vaccinated individuals in each month, and 95% CIs were calculated using the Poisson distribution. Participants were followed up from the date of vaccination until the occurrence of an influenza infection, death, or end of study period (31 March 2024).

(1) $$\begin{array}{l} Cumulative\;incidence\;of\;overall\;influenza\;infection\\ \;\;\;\;\;=\left(\frac{Number\;of\;new\;overall\;influenza\;infection\;cases\;}{Total\;number\;of\;individuals\;at\;risk}\right)\times 100 \end{array}$$

**Theorem 1.**  *The equation of cumulative incidence of overall influenza infection*.

The incidence rate of influenza infections was defined as the number of new influenza infection cases divided by the total person-time at risk, expressed per 100 person-months. Person-time was accumulated from the date of vaccination until the earliest of the following events: occurrence of an influenza infection, death, or the end of the follow-up period (31 March 2024). Ninety-five percent confidence intervals (CIs) for incidence rates were estimated assuming a Poisson distribution. The incidence rate of overall influenza infections was calculated using the following equation:

(2) $$\begin{array}{l} Incidence\;rate\;of\;overall\;influenza\;infection\\ \;\;\;\;\;=\left(\frac{Number\;of\;new\;overall\;influenza\;infection\;cases\;}{Total\;person-months}\right)\times 100 \end{array}$$

**Theorem 2.**  *The equation of incidence rate of overall influenza infection*.

The cumulative incidence of hospitalization in influenza cases was calculated as follows.

(3) $$\begin{array}{l} Cumulative\;incidence\;of\;hospitalization\;in\;influenza\;cases\\ \;\;\;\;\;=\left(\frac{Number\;of\;new\;hospitalization\;due\;to\;influenza\;infection\;cases\;}{Total\;number\;of\;influenza\;infected\;population\;at\;risk}\right)\times 100 \end{array}$$

**Theorem 3.**  *The equation of cumulative incidence of hospitalization in influenza cases*.

Subgroup analysis was conducted exclusively in infants aged 6 to 11 months, for whom a two-dose regimen is recommended and stratified by number of doses received (one or two). Additionally, the cumulative incidence of hospitalization in influenza cases was calculated by dividing the number of new influenza-related hospitalizations by the total number of influenza cases each month. A two-tailed *p* value < 0.05 was considered statistically significant. All statistical analyses were performed using SAS Enterprise Guide 9.4 (SAS Institute Inc., Cary, NC, USA).

## 3. Results

### 3.1. Demographics

A total of 333 participants (mean age: 13.2 months [SD: 8.9]; 53.1% male) were included in the safety set, and 247 participants (mean age: 12.8 months [SD: 9.0]; 54.7% male) were included in the ad hoc subset. In the safety set, 218 participants (65.5%) had a Pediatric Comorbidity Index (PCI) of 0, and 134 participants (40.2%) received one dose. Similarly, in the ad hoc subset, 156 participants (63.1%) had a PCI of 0, and 98 participants (39.7%) received one dose. Overall, most participants had a PCI score of 0 or 1, indicating low comorbidity levels within this pediatric population [9]. Overall, there were no significant differences in the baseline characteristics between the safety set and ad hoc subset. The demographic characteristics of the participants are summarized in Table 1.

### 3.2. Safety

Among 333 participants in the safety set, 181 (54.4%) participants reported at least one case of AE within 28 days post-vaccination. A total of 555 cases of AEs were recorded, with 327 (58.9%) cases categorized as mild, 219 (39.5%) cases as moderate, and 9 (1.6%) cases as severe. Additionally, 61 (18.3%) participants experienced ADRs and 6 (1.8%) participants experienced SAEs. None of the SAEs were considered related to the influenza vaccine, and all 6 participants were recovered (Table 2).

All reported solicited AEs were evaluated and classified as ADRs. For local solicited AE, induration was the most commonly reported, occurring in 19 (5.7%) participants, while irritability was the most frequently reported systemic solicited AE, observed in 23 (6.9%) participants (Table S1).

A subgroup analysis was performed to evaluate the incidence of AEs based on the number of doses received. The incidence of AEs was higher in two-dose group (63.3%, 126/199 participants) compared with the one-dose group (41.0%, 55/134 participants) (*p* < 0.001). Regarding the severity of AEs, 72.6% (90/124 cases) in the one-dose group and 55.0% (237/431 cases) in the two-dose group were classified as mild. Moderate AEs were observed in 26.6% (33/124 cases) and 43.2% (186/431 cases), respectively. Severe AEs were reported in 0.8% (1/124 cases) of the one-dose group and 1.9% (8/431 cases) of the two-dose group (Table S2).

A significant age disparity was observed between the groups: the one-dose group had an average age of 20.2 months, while the two-dose group averaged 8.4 months (*p* < 0.001, Table S3). This finding was anticipated, as local prescribing guidelines recommend that previously unvaccinated children under 9 years receive two doses 4 weeks apart. When the same follow-up period was applied for each dose of the vaccination in the two-dose group, no differences were noted in the overall AE incidence, with rates ranging from 39.7% (79/199 patients) to 44.7% (89/199 patients) across two sub-groups—the first dose group (Dose 1) and the second dose group (Dose 2)—and the severity distribution was similar, with approximately 98% of cases classified as mild or moderate (Table S4). Likewise, in infants aged 6 to 11 months, AE incidence ranged from 38.5% (62/161 patients) to 47.2% (76/161 patients), with no differences among analyses. Comparing the safety after each dose of vaccination during the same follow-up period, there was no difference in AE incidence among two groups and most AEs were mild or moderate in severity. The safety profile was well tolerated across two groups (Table S5).

### 3.3. Influenza Infections

**Figure 2:**
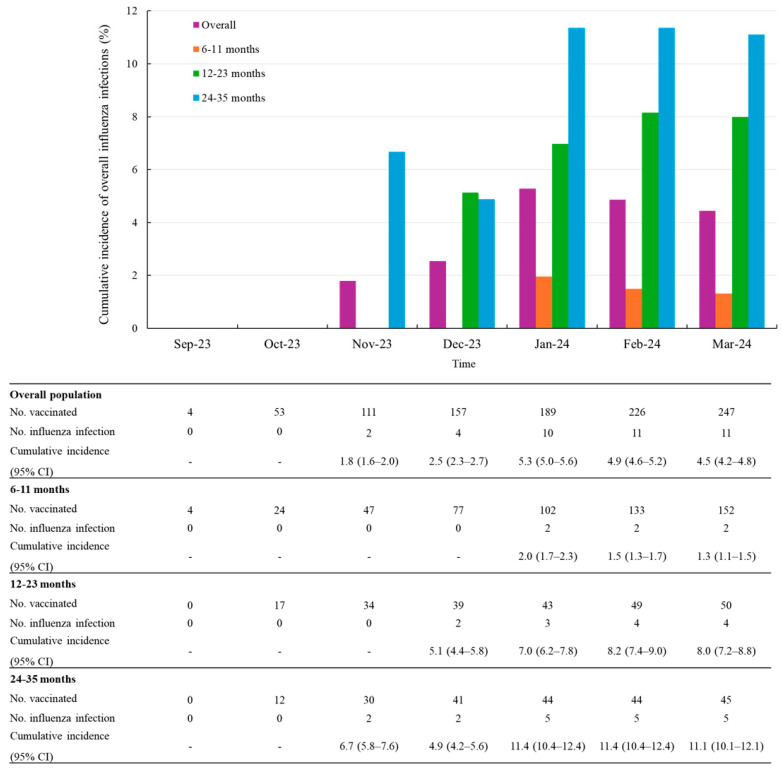
Cumulative incidence of overall influenza infections after vaccination stratified by age groups. Abbreviations: CI, confidence level.

Among the 247 participants in the ad hoc subset, influenza infections occurred in 11 participants after 14 days post-vaccination. The overall cumulative incidence of influenza infections was 4.5% (95% CI, 4.2–4.8) at the final follow-up time point as of March 2024. The cumulative incidence sharply increased between December 2023 and January 2024, rising from 2.5% (2.3–2.7) to 5.3% (5.0–5.6). Of the 11 influenza cases, the majority of influenza infections were reported between November 2023 and January 2024, with a particularly high frequency in January, accounting for 6 cases (approximately half of the total). The cumulative incidence of influenza infections varied by age group, with the highest incidence observed in children aged 24–35 months (11.1%, 5/45 patients) and the lowest in those aged 6–11 months (1.3%, 2/152 patients) (Figure 2).

During a mean follow-up of 3.38 months (SD 1.6), the incidence rate of influenza infection was 1.3 per 100 person-months (95% CI 0.7–2.4). When stratified by age group, the incidence rate was 0.4 per 100 person-months (95% CI 0.1–1.8) among infants aged 6–11 months, 2.0 (95% CI 0.8–5.3) among those aged 12–23 months, and the highest rate was observed in children aged 24–35 months, at 2.8 per 100 person-months (95% CI 1.2–6.7) Table 3 shows the incidence rate of influenza infection by time since last vaccination. Additionally, the median time from the first vaccination to influenza infection was 68 days.

To more precisely assess dose-related differences, we conducted a subgroup analysis exclusively in infants aged 6 to 11 months, for whom a two-dose regimen is clearly recommended. Among this group, the compliance rate for the two-dose schedule was 82.9% (126/152).

**Figure 3:**
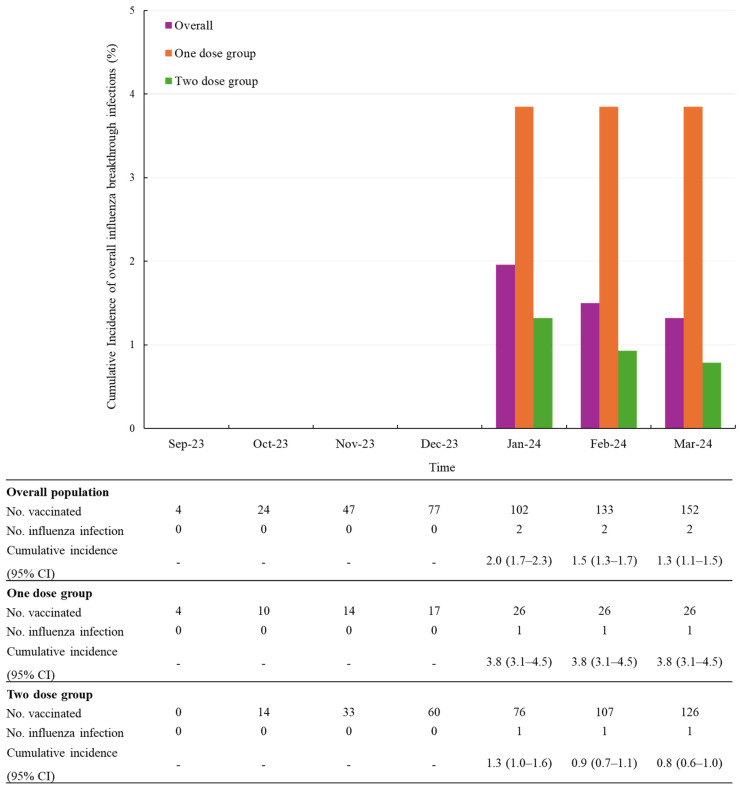
Cumulative incidence of overall influenza infections after vaccination, stratified by number of doses in infants aged 6 to 11 months. Abbreviations: CI, confidence level.

In this subgroup, the cumulative incidence of influenza infections was 0.8% (1/126) in the two-dose group compared to 3.8% (1/26) in the one-dose group (Figure 3).

During a mean follow-up of 3.0 months (SD 1.6), the overall incidence rate of influenza infection was 0.4 per 100 person-months (95% CI 0.1–1.8). Among children who received one dose, the incidence rate was 0.9 per 100 person-months (95% CI 0.1–6.6), whereas it was 0.3 per 100 person-months (95% CI 0.04–2.0) among those who received two doses (Table 4).

No influenza-related hospitalizations occurred among the vaccinated population. Of the two excluded participants, one case of influenza-related hospitalization was identified. This case occurred in a child who eventually completed the two-dose schedule, but the hospitalization took place after the first dose and before the second dose was administered.

## 4. Discussion

This prospective cohort and non-interventional study aimed to assess the safety profile within the scope of a conventional PMS study. During the 2023–2024 influenza season, subjects who participated in the PMS were additionally followed up until the end of the flu season to observe patterns of influenza infections.

The reported incidence rates of AEs in this study were 54.4% (181/333), and the incidence of SAEs was 1.8% (6/333). AEs were generally mild to moderate in severity. The incidence of AEs within 28 days of vaccination was similar regardless of the number of doses received or the order of doses. Additionally, approximately 98% of AEs were classified as mild or moderate. A notable aspect of this study was the use of an SMO during the 2023–2024 influenza season, which enabled active safety data collection through standardized and consistent methods by trained study staff. This structured approach contributed to the reliability of the collected safety data, addressing potential limitations in general PMS practices, such as inconsistent AE reporting and under-reporting. In general, the methodology for data collection is not clearly defined in the Korean PMS guidelines, and there are no comprehensive regulations or standards to ensure the collection of high-quality data. As a result, without proper oversight or standards, general PMS practices are sometimes at risk of being misused as a mere means of maintaining a product license, rather than as a tool for comprehensive safety profiling [11]. This cohort study was part of an active surveillance system. In contrast to passive surveillance, which relies on voluntary reporting and is prone to underreporting and bias, active surveillance monitors adverse events through safety follow-ups or medical record reviews. This approach provides more accurate and timely detection of adverse events, particularly among high-risk groups such as the pediatric population included in this study.

For the influenza infections after vaccination, the overall cumulative incidence during the study period was 4.5% (11/247), showing a positive trend with increasing age. This trend was also observed in the Korea Disease Control and Prevention Agency (KDCA) weekly sentinel surveillance report which reported an influenza-like illness (ILI) incidence. For indirect comparison of our study findings to KDCA reports, we aggregated weekly incidence of ILI into monthly incidence by calculating the average of weekly values within each month and converting the monthly incidence into cumulative incidence by summing the monthly values across all preceding months. According to KDCA reports, the cumulative incidence of ILI during the same period was 8.0% for infants aged 0–11 months and 18.3% for children aged 12–71 months. Furthermore, the influenza vaccination coverage for children aged 6 to 59 months under the immunization program was 82.5% during the 2023–2024 influenza season, with a market share of cell culture-derived inactivated quadrivalent influenza vaccine accounting for 21.6% [12].

The overall incidence rate of influenza infection during the study period was estimated at 1.3 per 100 person-months (95% CI 0.7–2.4). Age-stratified analysis indicated a higher incidence among children aged 12–23 and 24–35 months compared to those aged 6–11 months. This observed trend may be explained by increased exposure to environmental and social risk factors, such as participation in outdoor activities and higher frequency of interpersonal interactions in daycare settings, which are more prevalent in the older age groups [4]. These factors likely contributed to the elevated susceptibility to influenza infection among children aged 12–35 months relative to younger infants aged 6–11 months.

Given the non-interventional design of this study, participants received either a one-dose or two-dose vaccination regimen in accordance with routine clinical practice and national recommendations. To more accurately assess the impact of the two-dose regimen, the analysis was restricted to vaccine-naïve infants aged 6 to 11 months for whom a two-dose regimen is clearly recommended. The observed trend showed that the two-dose group had a lower cumulative incidence of influenza infections (0.8%, 1/126) compared to the one-dose group (3.8%, 1/26). This trend was similarly reflected in the incidence rates, with the two-dose group demonstrating a lower rate (0.3 per 100 person-months) relative to the one-dose group (0.9 per 100 person-months). This reinforces the importance of the two-dose recommendation for first-time influenza vaccination. The two-dose regimen for influenza vaccine-naïve children is supported by numerous studies that have shown reduced vaccine immunogenicity or effectiveness of vaccines with only one dose, and currently many countries recommend the two-dose regimen for those populations [13].

It is no doubt that one of ultimate goals of influenza immunization is to reduce the risk of hospitalization especially in vulnerable populations such as infants, elderly populations, people with certain medical conditions. In the ad hoc analysis, no cases of influenza-related hospitalization were identified. Given the single cohort study design in this analysis, again we also referenced one representative publication in regards to national claims data to provide context on the general severity of influenza cases in Korea over the past 10 years [14]. Since the NIP against influenza for children in Korea began in 2016, data from 2016 to 2020 were specifically quoted. During this period, the hospitalization rate among influenza cases in children under 5 years ranged from approximately 15% to 20%, with a vaccine uptake of around 61.5% to 83.5% [15,16]. This reference to national data provides a broader context for understanding the severity of influenza-related hospitalizations, though direct comparisons between cohorts with different vaccination coverage and infection burdens should be interpreted cautiously.

This study’s limitations must be acknowledged. It is a single-arm cohort study with the absence of a control group and a relatively small sample size made it difficult to derive more rigorous results. However, from a vaccine study methodology perspective, a high level of vaccination coverage could limit the traditional approach to evaluate the effectiveness of vaccines in real-world settings as it is hard to place the placebo group who were not vaccinated in the targeted season. To address this limitation, we conducted an indirect comparison using external representative data. However, it could be a second limitation of this study as indirect comparison with quoted references should be cautiously interpreted based on the limitation of the sources from different settings. To minimize the bias in indirect comparison with external data, we confirmed the influenza epidemiological pattern of our study period was similar to before the COVID-19 pandemic and assessed the quoted references for similarity, homogeneity, and suitability for indirect comparison.

## 5. Conclusions

Our study shows the tolerable safety profile and pattern of influenza infections in children aged 6–35 months in South Korea after SKYCellflu^®^ vaccination. Additionally, the two-dose vaccination regimen showed a trend of higher preventative effects against influenza infection, while maintaining a comparable safety profile 28 days post-vaccination. The role of continuous surveillance is critical, as seasonal influenza patterns fluctuate each year. Ongoing monitoring of both vaccine effectiveness and safety is essential to gain a more comprehensive understanding of vaccine safety, particularly in high-risk populations such as those included in this study. While our study had limitations in assessing vaccine effectiveness, the results of a test-negative design study with the same product provide valuable complementary data. These results further support the clinical value of cell-culture-derived influenza vaccines, and further studies with larger sample sizes or alternative study designs are needed to validate these findings across a broader pediatric population.

## Figures and Tables

**Table 1:** Demographics of study population.

Baseline Characteristics	Safety Set N = 333	Ad Hoc Subset N = 247
Mean age, months (SD)	13.2 (8.9)	12.8 (9.0)
Age group, n (%)		
6–11 months	195 (58.6)	152 (61.5)
12–23 months	74 (22.2)	50 (20.2)
24–35 months	64 (19.2)	45 (18.2)
Sex, n (%)		
Male	177 (53.1)	135 (54.7)
Female	156 (46.9)	112 (45.3)
Number of vaccine doses received, n (%)		
One dose	134 (40.2)	98 (39.7)
Two doses	199 (59.8)	149 (60.3)
Pediatric Comorbidity Index, means (SD)	0.5 (0.8)	0.5 (0.7)
Pediatric Comorbidity Index group, n (%)		
0	218 (65.5)	156 (63.1)
1	91 (27.3)	75 (30.4)
≥2	24 (7.2)	16 (6.5)

Abbreviations: SD, standard deviation.

**Table 2:** Safety analysis results within 28 days post-vaccination.

AEs	Safety Set N = 333
AE, n (%) [case]	181 (54.4) [555]
95% CI	48.8–59.8
Severity, case (%)	555 (100)
Mild	327 (58.9)
Moderate	219 (39.5)
Severe	9 (1.6)
ADR, n (%) [case]	61 (18.3) [129]
95% CI	14.3–22.9
SAE, n (%) [case]	6 (1.8) [6]
95% CI	0.7–3.9

Abbreviations: AE, adverse event; ADR, adverse drug reaction; CI, confidence level; SAE, severe adverse event.

**Table 3:** Incidence rate of influenza infections after vaccination stratified by age groups.

	Incidence Rate (95% CI) *						
Time Since Last Vaccination (Months)	1	2	3	4	5	6	7
Overall population	0.4 (0.1–3.0)	0.9 (0.3–2.4)	1.6 (0.9–3.0)	1.5 (0.8–2.7)	1.4 (0.8–2.5)	1.3 (0.7–2.4)	1.3 (0.7–2.4)
6–11 months	-	0.4 (0.1–2.7)	0.6 (0.1–2.3)	0.5 (0.1–1.9)	0.5 (0.1–1.8)	0.4 (0.1–1.8)	0.4 (0.1–1.8)
12–23 months	2 (0.3–14.4)	1.1 (0.2–7.6)	3.0 (1.1–8.0)	2.4 (0.9–6.4)	2.1 (0.8–5.6)	2.0 (0.8–5.3)	2.0 (0.8–5.3)
24–35 months	-	2.3 (0.6–9.1)	3.2 (1.2–8.4)	3.2 (1.3–7.6)	2.9 (1.2–6.9)	2.8 (1.2–6.7)	2.8 (1.2–6.7)

* Incidence rate per 100 person-months. Abbreviations: CI, confidence level.

**Table 4:** Incidence rate of influenza infections after vaccination stratified by number of doses in infants aged 6 to 11 months.

	Incidence Rate (95% CI) *						
Time Since Last Vaccination (Months)	1	2	3	4	5	6	7
Overall population	-	0.4 (0.1–2.7)	0.6 (0.1–2.3)	0.5 (0.1–1.9)	0.5 (0.1–1.8)	0.4 (0.1–1.8)	0.4 (0.1–1.8)
One dose group	-	1.9 (0.3–13.8)	1.4 (0.2–9.8)	1.2 (0.2–8.2)	1.0 (0.1–7.2)	0.9 (0.1–6.7)	0.9 (0.1–6.6)
Two dose group	-	-	0.4 (0.1–2.6)	0.3 (0.04–2.2)	0.3 (0.04–2.1)	0.3 (0.04–2.0)	0.3 (0.04–2.0)

* Incidence rate per 100 person-months. Abbreviations: CI, confidence level.

## Data Availability

The data presented in this study are available on request from the corresponding author due to participant privacy policy.

## Supplementary Materials

The following supporting information can be downloaded at: https://www.mdpi.com/article/10.3390/vaccines13050501/s1, Table S1: Incidence of solicited adverse events; Table S2: Incidence of adverse events stratified by the number of doses; Table S3: Baseline characteristics of study population stratified by the number of doses; Table S4: Incidence of adverse events stratified by vaccine dose with a 28-day post-vaccination period (Safety set); Table S5: Incidence of adverse events stratified by vaccine dose in infants aged 6–11 months with a 28-day post-vaccination period.

## Abbreviations

The following abbreviations are used in this manuscript:

NIP	national immunization program
MFDS	Ministry of Food and Drug Safety
PMS	post-marketing surveillance
AE	adverse events
EMR	electronic medical record
SMO	site management organization
IRB	Institutional Review Board
ADR	adverse drug reactions
SAE	serious adverse events
SD	standard deviation
CI	confidence intervals
KDCA	Korea Disease Control and Prevention Agency
ILINet	influenza-like illness surveillance network
