# Supplementary material for: Safety and Influenza Infections in Children Aged 6–35 Months Receiving Cell Culture-Derived Inactivated Quadrivalent Influenza Vaccine During the 2023–2024 Influenza Season in South Korea
Source: Vaccines (Basel). 2025 May 8;13(5):501. doi: 10.3390/vaccines13050501 (PMC12115390; doi:10.3390/vaccines13050501)
Supplement: Supplementary file 1 [file vaccines-13-00501-s001.zip › vaccines-3582003-supplementary.pdf]

Table S1. Incidence of solicited adverse events

Solicited AEs	Safety set N=333		
	**N (%)**	**95% CI**	**Case**
Local solicited AEs			
Pain/Tenderness	13 (3.9)	2.1-6.6	19
Erythema	18 (5.4)	3.2-8.4	19
Oedema	10 (3.0)	1.4-5.5	11
Induration	19 (5.7)	3.5-8.8	19
Systemic solicited AEs			
Irritablity	23 (6.9)	4.4-10.2	25
Pyrexia	10 (3.0)	1.4-5.5	11
Somnolence	10 (3.0)	1.4-5.5	10

Abbreviation: AE, adverse events; CI, confidence interval.

Table S2. Incidence of adverse events stratified by the number of doses

AEs	One dose N=134	Two doses N=199	*p*
AE, n (%) [Case]	55 (41.0) [124]	126 (63.3) [431]	<0.001
95% CI	32.6-49.9	56.2-70.0	
Severity, case (%)			
Mild	90 (72.6)	237 (55.0)	
Moderate	33 (26.6)	186 (43.2)	
Severe	1 (0.8)	8 (1.8)	

Abbreviation: AE, adverse events; CI, confidence interval.

Table S3. Baseline characteristics of study population stratified by the number of doses

Baseline characteristics	Safety set N=333		*p*
	**One dose N=134**	**Two doses N=199**	
Mean age, months (SD)	20.2 (9.6)	8.4 (4.0)	<0.001
Age group, n (%)			<0.001
6 – 11 months	34 (25.4)	161 (80.9)	
12 – 23 months	39 (29.1)	35 (17.6)	
24 – 35 months	61 (45.5)	3 (1.5)	
Sex, n (%)			0.163
Male	65 (48.5)	112 (56.3)	
Female	69 (51.5)	87 (43.7)	
Pediatric Comorbidity Index, means (SD)	0.6 (0.7)	0.4 (0.8)	0.005
Pediatric Comorbidity Index group, n (%)			<0.001
0	67 (50.0)	151 (75.9)	
1	58 (43.3)	33 (16.6)	
≥2	9 (6.7)	15 (7.5)	

Abbreviation: SD, standard deviation

Table S4. Incidence of adverse events stratified by vaccine dose with a 28-day post-vaccination period (Safety set)

AEs	One dose N=134	Two doses, N=199	
		Dose1	Dose2
AE, n (%) [Case]	55 (41.0) [124]	89 (44.7) [247]	79 (39.7) [184]
95% CI	32.6-49.9	37.7-51.9	32.9-46.9
Severity, case (%)			
Mild	90 (72.6)	133 (53.8)	104 (56.5)
Moderate	33 (26.6)	109 (44.1)	77 (41.8)
Severe	1 (0.8)	5 (2.1)	3 (1.7)

Abbreviation: AE, adverse events; CI, confidence interval.

Table S5. Incidence of adverse events stratified by vaccine dose in infants aged 6 -11 months with a 28-day post-vaccination period

AEs	One dose N=34	Two doses, N=161	
		Dose1	Dose2
AE, n (%) [Case]	14 (41.0) [25]	76 (47.2) [223]	62 (38.5) [148]
95% CI	26.4-57.8	39.3-55.2	31.0-46.5
Severity, case (%)			
Mild	18 (72.0)	123 (55.2)	84 (56.8)
Moderate	7 (28.0)	96 (43.0)	62 (41.9)
Severe	0	4 (1.8)	2 (1.3)

Abbreviation: AE, adverse events; CI, confidence interval.
